# Fasting blood glucose trajectories and atherosclerosis risk: a longitudinal cohort study with threshold analysis in Chongqing, China

**DOI:** 10.1186/s41043-025-01193-7

**Published:** 2026-03-05

**Authors:** Na Li, Chun-Mei Fei, Feng Tang, Xian-Shu Lin, Bing-Rui Yang, Jun Guo, Li-An-Sheng Wu, Yin-Yin Xia, Chuan Zhang, Li Xu

**Affiliations:** 1 Department of Health Management Center, The First Affiliated Hospital of Chongqing Medical University, Chongqing, 400016 China; 2 School of Public Health, Chongqing Medical University, Chongqing, 400016 China; 3 Department of Cardiology, The First Affiliated Hospital of Chongqing Medical University, Chongqing, 400016 China; 4 Department of Nursing, The First Affiliated Hospital of Chongqing Medical University, Chongqing, 400016 China

**Keywords:** Fasting blood glucose, Atherosclerosis risk, Group-based trajectory model, Threshold analysis, Prediabetes mellitus, Longitudinal study

## Abstract

**Background:**

Atherosclerosis, the primary pathological basis of cardiovascular diseases, exhibits a strong association with glucose metabolism dysregulation. While cross-sectional studies have linked fasting blood glucose (FBG) to atherosclerosis risk, the dose-response relationship and threshold characteristics of long-term FBG trajectories remain poorly characterized. This retrospective cohort study aimed to investigate longitudinal FBG trajectory patterns and their associations with atherosclerosis risk prevalence, incidence, and recovery in Chongqing, China, while also identifying population-specific risk thresholds.

**Methods:**

Based on the three-year longitudinal follow-up data collected annually from 2017 to 2019, a population-based trajectory model (GBTM) was adopted to identify the dynamic trajectory of FBG. The association between FBG and atherosclerosis risk was analyzed using multivariable logistic regression. Restricted cubic splines (RCS) were used to assess the non-linear relationship between FBG and atherosclerosis risk and to determine risk thresholds. Confounding factors such as age, sex, body mass index (BMI), blood pressure, and lipids were adjusted for in the regression models, and subgroup analyses were performed to examine the interactions of age, sex, and BMI.

**Results:**

Longitudinal analysis showed that compared with the Trajectory Normal Glucose Regulation (NGR) group, the Trajectory Prediabetes Mellitus group (Pre-DM) group had significantly higher prevalence (*OR*: 2.02, 95% *CI*: 1.63–2.51) and incidence (*OR*: 1.66, 95% *CI*: 1.15–2.39) of atherosclerosis risk. The Trajectory Pre-DM group also had a significantly lower likelihood of atherosclerosis risk recovery than the Trajectory NGR group (*OR*: 0.55, 95% *CI*: 0.39–0.79). Dose-response analysis revealed a non-linear association between FBG and atherosclerosis risk prevalence, with a risk threshold at 5.10 mmol/L. This suggests that the atherosclerosis risk threshold in Chongqing is significantly lower than the international prediabetes standard of 5.60 mmol/L. Subgroup analyses showed sex and age differences, with lower thresholds in women and younger individuals.

**Conclusions:**

Long-term elevation of FBG was associated with increased atherosclerosis risk. The study suggests that intervention strategies should be based on dynamic blood glucose trajectories and population-specific thresholds, especially lower thresholds for women and younger individuals. This study provides evidence-based support for regional atherosclerosis risk prevention and control.

**Supplementary Information:**

The online version contains supplementary material available at 10.1186/s41043-025-01193-7.

## Introduction

 Atherosclerosis is a progressive disease of medium- and large-sized arteries, characterized by endothelial injury and chronic inflammatory responses [1, 2]. In the early stages, the disease is marked by subendothelial lipid accumulation, which gradually develops into plaques with fibrous caps [3]. The continued growth of these plaques can lead to narrowing of vascular lumen, resulting in local tissue ischemia, hypoxia, and metabolic disturbances [4, 5]. If the fibrous cap ruptures or the plaque surface undergoes erosion, subendothelial components become exposed, triggering platelet aggregation and activation of the coagulation cascade [6, 7]. This process is a direct precipitating factor for conditions such as acute coronary syndrome and ischemic stroke [8]. According to the *Summary of the Report on Cardiovascular Health and Diseases in China 2023* [9], the burden of atherosclerosis related diseases in China continue to worsen. Cardiovascular disease has remained the leading cause of death among both urban and rural residents for several consecutive years [10]. Currently, an estimated 11.39 million people are living with coronary heart disease, and 13.00 million with stroke [10]. As the primary pathological basis of life-threatening and disabling conditions such as coronary heart disease and stroke, atherosclerosis has posed a serious threat to public health and placed a substantial burden on healthcare resources [11, 12].

Fasting blood glucose (FBG) is crucial for monitoring glycemic status, enabling the early detection of prediabetes and the assessment of cardiovascular risk [13]. Abnormally elevated FBG may accelerate the pathological progression of atherosclerosis by inducing insulin resistance and promoting chronic inflammatory responses, leading to increased plasma triglycerides (TG) and decreased high-density lipoprotein cholesterol (HDL-C) levels [14, 15]. The atherogenic index of plasma (AIP), a novel biomarker reflecting the pro-atherogenic potential of the lipid profile, was first proposed by Dobiásová et al. in 2001 [16, 17]. It is calculated as log_10_ (TG/HDL-C) [18, 19]. Compared with traditional lipid parameters, AIP more accurately indicates the imbalance of lipid micro-environment between atherosclerosis risk promoting and anti-atherosclerosis risk [20–22]. AIP is directly related to the degree of vascular endothelial inflammation, plaque stability, and other core pathological features of atherosclerosis risk [23]. Its low cost and reliance on routine lipid tests make it highly accessible and reproducible [24].

Although existing studies have confirmed a non-linear relationship between FBG and cardiovascular disease events, and that either too high or too low FBG levels can increase the risk of cardiovascular disease events [25, 26]. However, most of these studies rely on cross-sectional designs or single time point FBG measurements, failing to capture the dynamic changes in glucose metabolism and their cumulative effects on atherosclerosis risk [27, 28]. In addition, the population-specific thresholds of FBG associated with atherosclerosis risk have not yet been determined, especially in regions with unique genetic, dietary, and lifestyle characteristics [29]. This study, conducted in the southwestern region of China, establishes a cohort of the residents in Chongqing with the following objectives: (1) To investigate the associations between the longitudinal trajectories of FBG and the prevalence, incidence, and recovery of atherosclerosis risk; (2) To develop an atherosclerosis risk prediction model based on dynamic glucose monitoring; (3) To establish FBG intervention thresholds applicable to the local residents. This study provides evidence-based support for optimizing the prevention strategies of atherosclerosis risk and has important public health significance for improving the early identification of high-risk populations and the regional management of chronic diseases.

## Materials and methods

### Study population

**Fig. 1:**
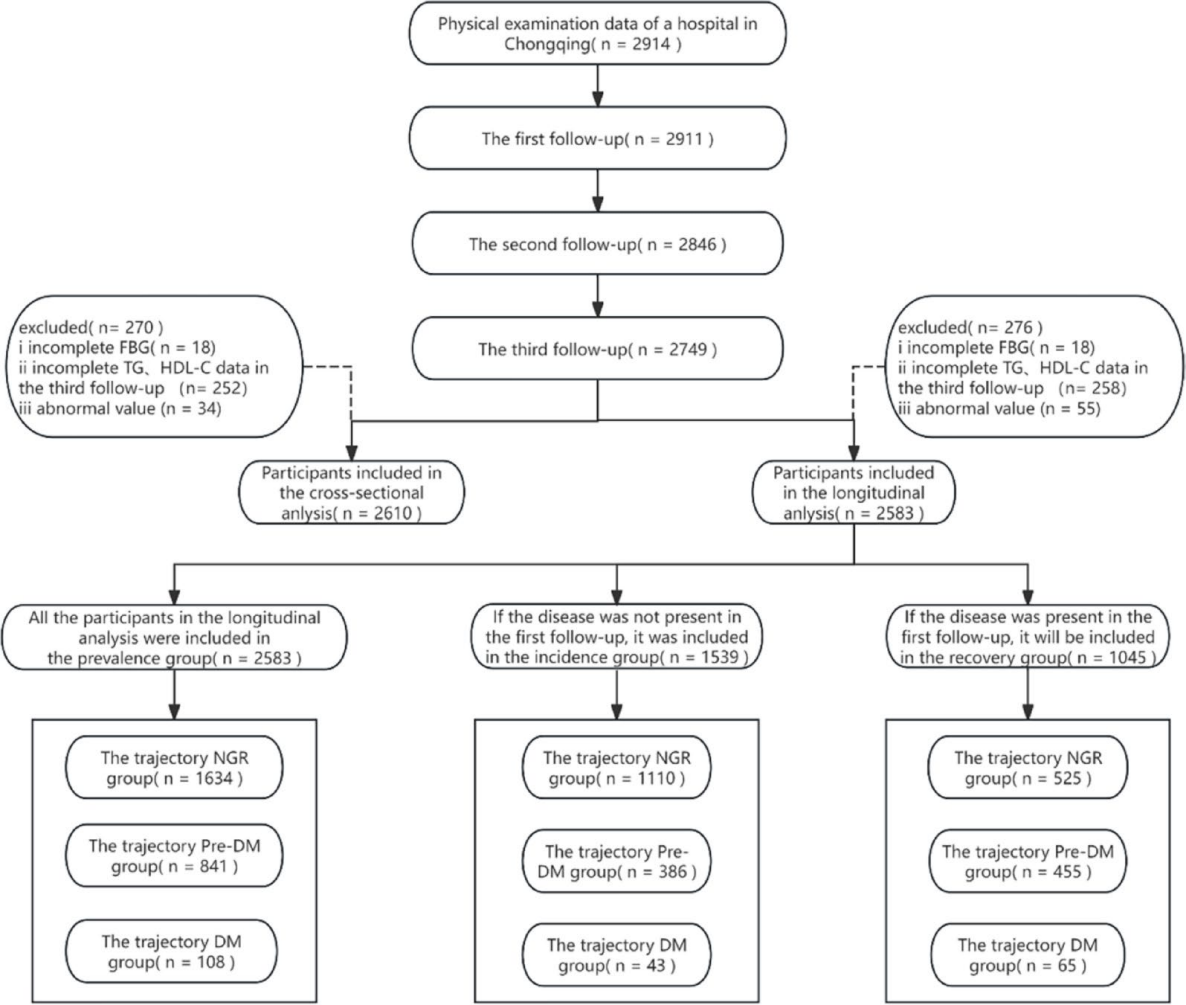
Flowchart for Inclusion/Exclusion of Study Participants

Data for this study were derived from the health check-up population at the outpatient health management center of JinShan Hospital, the First Affiliated Hospital of Chongqing Medical University. A total of 2,914 participants were initially recruited in this study. The health examination data of 2017, 2018 and 2019 were selected for follow-up analysis annually for a total of three times. Individuals who participated The 2019 health check-up and had complete data on FBG, TG, and HDL-C were included in the cross-sectional analysis. For the longitudinal analysis, participants with complete measurements of FBG, TG, and HDL-C across all three time points were selected. Outliers were excluded during data processing (Fig. 1). During the data cleaning stage, we excluded participants who presented physiologically impossible values in the continuous variables. After exclusions, 2,610 participants were eligible for the cross-sectional analysis and 2,583 for the longitudinal analysis. A post-hoc power analysis was performed using G*Power 3.1.9.7, which confirmed that our sample size provided a statistical power > 0.99 (Fig. S1).

This research was approved by the Ethics Committee of the First Affiliated Hospital of Chongqing Medical University (2022-K122). The data collection and usage complied with the principles of medical ethics and the requirements of the Declaration of Helsinki, without any risk or damage to health, security or privacy for the participants. Informed consent was obtained from all the participants prior to the enrollment of this study.

### Data collection and measurements

Demographic information including age and sex, as well as lifestyle factors, was collected using standardized questionnaires. Anthropometric measurements, including height (m), weight (kg), and waist circumference (cm), were obtained by trained personnel using calibrated instruments. Body mass index (BMI) was calculated as weight divided by height in meters squared (kg/m²) [30]. Blood pressure (mmHg) was measured three times on the left arm using electronic sphygmomanometers after a 15-minute rest, and the average of the three readings was recorded.

Fasting venous blood samples were collected after an overnight fast of at least 8 h. Biochemical parameters, including FBG (mmol/L), total cholesterol (TC, mmol/L), TG (mmol/L), HDL-C (mmol/L), low-density lipoprotein cholesterol (LDL-C, mmol/L), uric acid (UA, µmol/L), and serum creatinine (Cr, µmol/L), were measured using standardized enzymatic or colorimetric methods on automated analyzers, adhering to established clinical laboratory guidelines [31, 32]. The AIP was calculated as log_10_(TG/HDL-C). Hypertension was defined as having a systolic blood pressure and/or diastolic blood pressure ≥ 140/90 mmHg or using antihypertensive medication. According to the American Diabetes Association (ADA) standards, diabetes mellitus (DM) was defined as an FBG ≥ 7.0 mmol/L or self-reported physician diagnosis of diabetes. Normal glucose regulation (NGR) was defined as an FBG < 5.6 mmol/L [33]. Prediabetes mellitus (Pre-DM) was defined as an FBG between the thresholds for normal glucose regulation and diabetes. Dyslipidemia was defined as meeting any of the following conditions: high TG (TG ≥ 2.26 mmol/L), high TC (TC ≥ 6.22 mmol/L), high LDL-C (LDL-C ≥ 4.14 mmol/L), or low HDL-C (HDL-C < 1.04 mmol/L)[34].

### Assessment of outcomes

The primary outcomes of this study were the prevalence, incidence, and recovery of atherosclerosis risk. Given that imaging-based vascular assessments such as carotid intima-media thickness and coronary calcium scoring are not routinely available in large-scale health check-up datasets, AIP was selected as a validated, cost-effective, and reproducible surrogate indicator of atherosclerosis risk [24, 35]. Atherosclerosis risk was defined as the AIP ≥ 0.1 [17, 36, 37]. Atherosclerosis risk recovery was defined as participants whose AIP was ≥ 0.1 at the baseline in 2017, and whose AIP decreased to < 0.1 at the third follow-up in 2019. In this study, the presence of illness, the incidence of disease, and the recovery were defined as dichotomous variables labeled as 1 (presence of illness, incidence of disease, and recovery) and 0 (no illness, no incidence of disease, and no recovery), respectively.

### Statistical analysis

Data analyses were performed using RStudio (2024.12.0 + 467) and SPSS version 20, with all statistical tests being two-sided and a *P* value < 0.05 considered statistically significant. Normally distributed continuous variables are expressed as mean ± standard deviation (x̄ ± s) and compared using one-way ANOVA; skewed continuous variables are presented as medians (interquartile ranges, IQR) and compared using the Kruskal-Wallis H test; categorical variables are expressed as frequencies and percentages and compared using the chi-square test. Multiple comparison correction was performed using Bonferroni. In the cross-sectional analysis, multifactor logistic regression was used to determine the association between atherosclerosis risk and different glycemic statuses. In the longitudinal analysis, a group-based trajectory model (GBTM) was used to identify long-term FBG trajectories by grouping participants with similar FBG change patterns over three years.

GBTM, also known as latent class growth modeling or semi-parametric group-based modeling, was introduced by Nagin [38]. This method does not account for within-group variation and assumes that individuals within a given group follow an identical trajectory over time. It is a statistical approach designed to identify subgroups of individuals who share similar developmental trajectories by analyzing temporal changes in behaviors or outcomes [39]. As an application of finite mixture modeling, GBTM is particularly suited for the analysis of longitudinal data and is capable of revealing dynamic patterns of individual change in specific outcomes or behaviors [40]. The optimal number of trajectory groups and model selection were based on the following criteria: (1) relatively low Bayesian Information Criterion (BIC); (2) lower Akaike Information Criterion (AIC); (3) entropy value > 0.70; (4) proportion of each group > 3%; and (5) average posterior probability of assignment (APPA) > 0.7 [41]. First, univariate logistic regression was conducted to identify potential covariates. Second, multifactor logistic regression was used to assess the association between FBG trajectories and atherosclerosis risk. Three models were fitted: Model 1 estimated crude ORs without adjustment; Model 2 adjusted for age and sex; Model 3 further adjusted for BMI, SBP, DBP, heart rate, waistline, hemoglobin (HGB), UA, and Cr. Then, restricted cubic splines (RCS) were used to visualize the association between FBG levels and atherosclerosis risk, and to determine the prognostic value of FBG trajectories on atherosclerosis risk. RCS analysis takes the median of FBG as the reference point and determines how many nodes to select to fit the nonlinear relationship based on the AIC value. Additionally, the potential heterogeneity related to common phenotypes (including BMI, age, and sex) in the transition of atherosclerosis status was explored. BMI classification was based on recommendations by the Chinese Obesity Working Group, and age grouping followed World Health Organization (WHO) standards; differences between groups were compared using likelihood ratio tests.

## Results

### Cross-sectional analysis

A total of 2,610 participants were included in this study, among whom 66.86% were male. The median age was 44.00, and the median AIP was 0.02. The participants were divided into three groups according to their glycemic status: the normal glucose regulation group (NGR Group), the prediabetes mellitus group (Pre-DM Group), and the diabetes mellitus group (DM Group) (Table 1). Compared with the NGR group, participants who were male, older in age, and had a higher prevalence of hypertension and dyslipidemia were more likely to have abnormal blood glucose. In addition, the levels of BMI, SBP, DBP, waistline, AIP, TG, UA, Cr, and HGB were all positively correlated with the increase in blood glucose levels, while HDL-C was negatively correlated (*P* < 0.001).

**Table 1:** Subject characteristics classified by blood glucose status at the third follow-up in 2019 (*n* = 2610)

Character	Blood glucose status			*P-*value
	NGR group	Pre-DM group	DM group	
N (%)	1737 (66.55)	745 (28.54)	128 (4.90)	
Male, n (%)	1060 (61.02)	576 (77.32) ^a^	109 (85.16) ^b^	< 0.001
Age, years	36.00 (33.00, 52.00)	53.00 (45.00, 61.00) ^a^	55.50 (46.00, 63.00) ^b^	< 0.001
BMI, kg/m^2^	23.23 (21.09, 25.43)	24.91 (23.05, 26.87) ^a^	25.21 (23.46, 27.85) ^b^	< 0.001
SBP, mmHg	119.00 (109.00, 130.00)	130.00 (120.00, 140.00) ^a^	131.00 (120.75, 147.00) ^b^	< 0.001
DBP, mmHg	72.00 (65.00, 80.00)	79.00 (72.00, 87.00) ^a^	80.00 (71.00, 88.00) ^b^	< 0.001
Heart rate, bpm	78.00 (70.00, 88.00)	78.00 (70.00, 88.00)	77.00 (69.00, 85.00)	0.460
Waistline, cm	82.00 (74.00, 90.00)	88.00 (83.00, 93.00) ^a^	91.00 (84.00, 95.00) ^b, c^	< 0.001
TC, mmol/L	4.98 (4.37, 5.62)	5.16 (4.51, 5.79) ^a^	5.15 (4.37, 5.70)	< 0.001
TG, mmol/L	1.22 (0.83, 1.81)	1.71 (1.21, 2.44) ^a^	1.84 (1.24, 2.81) ^b^	< 0.001
HDL-C, mmol/L	1.34 (1.14, 1.59)	1.24 (1.08, 1.43) ^a^	1.19 (1.04, 1.40) ^b^	< 0.001
LDL-C, mmol/L	2.74 (2.27, 3.27)	2.84 (2.26, 3.37)	2.68 (2.08, 3.32)	0.124
AIP	−0.05 (−0.26, 0.19)	0.14 (−0.06, 0.33) ^a^	0.18 (−0.03, 0.42) ^b^	< 0.001
FBG, mmol/L	5.00 (4.70, 5.20)	6.00 (5.80, 6.30) ^a^	7.40 (7.10, 8.03) ^b, c^	< 0.001
HGB, mmol/L	151.00 (138.00, 161.00)	154.00 (146.00, 163.00) ^a^	157.00 (145.75, 164.25) ^b^	< 0.001
Cr, µmol/L	72.00 (59.00, 83.00)	76.00 (66.00, 84.00) ^a^	73.50 (65.00, 83.00)	< 0.001
UA, µmol/L	364.00 (298.00, 437.00)	391.00 (329.00, 452.00) ^a^	382.00 (330.75, 462.50) ^b^	< 0.001
Hypertension, n (%)	231 (13.30)	218 (29.26) ^a^	52 (40.62) ^b, c^	< 0.001
Dyslipidemia, n (%)	530 (30.51)	342 (45.91) ^a^	69 (53.91) ^b^	< 0.001

AIP, atherogenic index of plasma, is calculated by the formula: AIP = log₁₀ [TG (mmol/L)/HDL-C (mmol/L)]

^a^ indicates that the Pre-DM group is different from the NGR group;^b^ indicates that there are differences between DM group and NGR group.^c^ indicates that the Pre-DM group is differentfrom the DM group

Using the NGR group as the reference group, in both the unadjusted model and the model adjusted for age and sex, the AIP of the participants was significantly positively correlated with the Pre-DM group and the DM group (*P* < 0.05). After further adjustment for BMI, SBP, DBP, heart rate, waistline, LDL-C, HGB, UA, and Cr, the results were consistent with the above findings. The AIP was positively correlated with the Pre-DM group (odds ratio [*OR*]: 1.85, 95% *CI*: 1.49–2.30) and also positively correlated with the DM group (*OR*: 2.02, 95% *CI*:1.31–3.12), with *P* < 0.05 (Table 2).

**Table 2:** The correlation between blood glucose status and AIP in the third follow-up analysis in 2019

	Unadjusted OR (95% CI)	Model 1 OR (95% CI)	Model 2 OR (95% CI)
NGR group	Reference	Reference	Reference
Pre-DM group	2.42 (2.02–2.88)	2.32 (1.90–2.84)	1.85 (1.49–2.30)
DM group	3.08 (2.13–4.45)	2.72 (1.84–4.04)	2.02 (1.31–3.12)

Model 1, logistic regression adjusted for sex and age

Model 2, logistic regression adjusted for sex and age, BMI, SBP, DBP, heart rate, waistline, LDL-C, HGB, Cr, UA

### Longitudinal analysis

**Fig. 2:**
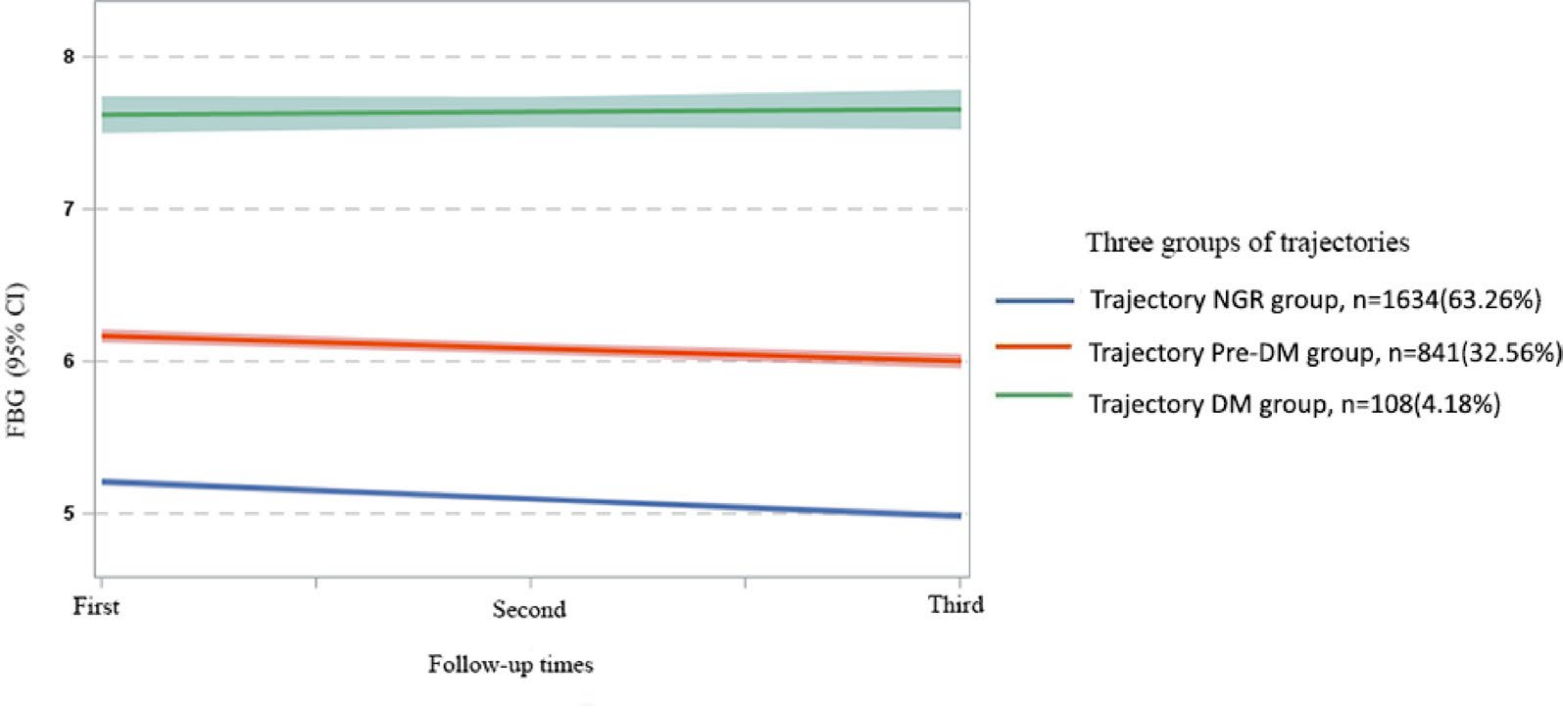
Trajectory of different FBG groups over time

FBG trajectory fitting. FBG was used as the dependent variable to fit a group-based trajectory model for FBG levels. The optimal number of trajectory groups and the best-fit model were determined based on the GBTM criteria (Table S1). According to the model fitting criteria, the optimal model in this study was the one with three trajectory groups. This model had the BIC value closest to 0 (−8445.00), the AIC value of −8413.54, an entropy value of 0.85 > 0.70, a minimum value of the APPA of 88.55 > 0.70, and the proportion of each category was > 3%. After fitting, the optimal model divided the FBG cohort into three groups, which based on the curve characteristics and the ADA criteria for diabetes diagnosis, were named as follows: Trajectory Normal Glucose Regulation group (1634 individuals, 63.26%), Trajectory Prediabetes Mellitus group (841 individuals, 32.56%), and Trajectory Diabetes Mellitus group (108 individuals, 4.18%). The trajectories of the Normal Glucose Regulation and Prediabetes groups were similar, both showing a downward trend over time, while the Diabetes group exhibited increasing FBG levels over time (Fig. 2).

A total of 2,583 individuals underwent health check-ups for this study, comprising 1,729 males (66.94%) and 854 females (33.06%), with a median age of 42.00 years. Based on FBG trajectories, participants were categorized into three groups. Compared to the Trajectory NGR group, participants in the Trajectory Pre-DM and Trajectory DM groups were more likely to be older males with a higher prevalence of hypertension and dyslipidemia. Moreover, their BMI, SBP, DBP, heart rate, waistline, TC, TG, HDL-C, LDL-C, AIP, HGB, UA, and Cr levels were significantly elevated, as shown in Table 3. During the third follow-up, the Trajectory Pre-DM and Trajectory DM groups continued to exhibit more pronounced metabolic abnormalities. Notably, the likelihood of atherosclerosis risk recovery was significantly lower in the Trajectory Pre-DM group than in the Trajectory NGR group, as presented in Table 4.

**Table 3:** Baseline characteristics of participants grouped by FBG trajectory in 2017 (*n* = 2583)

Character	FBG trajectory grouping			*P-*value
	Trajectory NGR group	Trajectory Pre-DM group	Trajectory DM group	
N (%)	1634 (63.26)	841 (32.56)	108 (4.18)	
Prevalence, n (%)	525 (32.13)	455 (54.10) ^a^	65(60.19) ^b^	< 0.001
Male, n (%)	982 (60.10)	651 (77.41) ^a^	96 (88.89) ^b, c^	< 0.001
Age, years	34.00 (31.00, 49.00)	51.00 (44.00, 59.00) ^a^	55.50 (47.75, 62.00) ^b^	< 0.001
BMI, kg/m^2^	22.78 (20.67, 24.98)	24.61 (22.76, 26.45) ^a^	24.96 (23.21, 26.72) ^b^	< 0.001
SBP, mmHg	119.00 (110.00, 130.00)	129.00 (119.00, 143.00) ^a^	132.00 (123.00, 143.25) ^b^	< 0.001
DBP, mmHg	73.00 (66.00, 80.00)	79.00 (72.00, 87.00) ^a^	80.00 (72.75, 88.00) ^b^	< 0.001
Heart rate, bpm	78.00 (70.00, 87.00)	77.00 (69.00, 86.00)	74.00 (68.00, 85.25)	0.027
Waistline, cm	81.00 (72.00, 88.00)	87.00 (81.00, 92.00) ^a^	88.00 (83.00, 93.00) ^b^	< 0.001
TC, mmol/L	5.00 (4.36, 5.61)	5.18 (4.58, 5.88) ^a^	5.22 (4.45, 5.81)	< 0.001
TG, mmol/L	1.18 (0.81, 1.78)	1.74 (1.17, 2.51) ^a^	1.70 (1.24, 2.82) ^b^	< 0.001
HDL-C, mmol/L	1.34 (1.14, 1.57)	1.23 (1.06, 1.44) ^a^	1.18 (1.00, 1.34) ^b^	< 0.001
LDL-C, mmol/L	2.90 (2.34, 3.47)	3.17 (2.65, 3.70) ^a^	3.18 (2.61, 3.60)	< 0.001
AIP	−0.06 (−0.27, 0.17)	0.14 (−0.06, 0.35) ^a^	0.18 (−0.04, 0.38) ^b^	< 0.001
FBG, mmol/L	5.20 (4.90, 5.40)	6.10 (5.80, 6.40) ^a^	7.10 (6.70, 8.00) ^b, c^	< 0.001
HGB, mmol/L	152.00 (139.00, 162.00)	155.00 (146.00, 163.00) ^a^	156.00 (147.00, 163.00) ^b^	< 0.001
Cr, µmol/L	71.00 (58.00, 81.00)	76.00 (66.00, 85.00) ^a^	74.00 (65.00, 84.25) ^b^	< 0.001
UA, µmol/L	348.00 (278.00, 417.00)	379.00 (316.00, 446.00) ^a^	379.00 (320.75, 443.00) ^b^	< 0.001
Hypertension, n (%)	236 (14.44)	280 (33.29) ^a^	42 (38.89) ^b^	< 0.001
Dyslipidemia, n (%)	490 (29.99)	406 (48.28) ^a^	57 (52.78) ^b^	< 0.001

^a^ indicates that the Trajectory Pre-DM group is different from the Trajectory NGR group; ^b^ indicates that there are differences between Trajectory DM group and Trajectory NGR group. ^c^ indicates that the Trajectory Pre-DM group is different from the Trajectory DM group

**Table 4:** Characteristics of participants grouped according to FBG trajectory in 2019 (*n* = 2583)

Character	FBG trajectory grouping			*P-*value
	Trajectory NGR group	Trajectory Pre-DM group	Trajectory DM group	
N (%)	1634 (63.26)	841 (32.56)	108 (4.18)	
Prevalence, n (%)	535 (32.74)	468 (55.65) ^a^	57 (52.78) ^b^	< 0.001
Incidence, n (%)	133 (11.99)	92 (23.83)	12 (27.91)	< 0.001
Recovery, n (%)	123 (23.43)	79 (17.36)	20 (30.77) ^b, c^	0.010
Male, n (%)	982 (60.10)	651 (77.41) ^a^	96 (88.89) ^b, c^	< 0.001
Age, years	36.00 (33.00, 51.00)	53.00 (46.00, 61.00) ^a^	57.00 (49.00, 64.00) ^b^	< 0.001
BMI, kg/m^2^	23.18 (20.96, 25.38)	24.93 (23.14, 26.91) ^a^	24.80 (23.15, 27.04) ^b^	< 0.001
SBP, mmHg	119.00 (109.00, 129.00)	130.00 (119.00, 141.00) ^a^	131.00 (120.75, 144.00) ^b^	< 0.001
DBP, mmHg	72.00 (65.00, 79.00)	78.00 (71.00, 86.00) ^a^	78.50 (71.00, 88.00) ^b^	< 0.001
Heart rate, bpm	79.00 (70.00, 88.00)	77.00 (69.00, 87.00)	77.50 (67.75, 87.00)	0.085
Waistline, cm	82.00 (73.00, 89.00)	88.00 (82.00, 93.00) ^a^	90.00 (84.00, 94.00) ^b^	< 0.001
TC, mmol/L	4.97 (4.35, 5.59)	5.19 (4.58, 5.82) ^a^	4.90 (4.06, 5.63) ^c^	< 0.001
TG, mmol/L	1.20 (0.82, 1.77)	1.70 (1.23, 2.48) ^a^	1.64 (1.14, 2.38) ^b^	< 0.001
HDL-C, mmol/L	1.35 (1.14, 1.59)	1.24 (1.08, 1.43) ^a^	1.21 (1.03, 1.42) ^b^	< 0.001
LDL-C, mmol/L	2.74 (2.27, 3.25)	2.87 (2.30, 3.41) ^a^	2.59 (2.03, 3.19) ^c^	0.001
AIP	−0.05 (−0.26, 0.18)	0.14 (−0.05, 0.33) ^a^	0.12 (−0.06, 0.35) ^b^	< 0.001
FBG, mmol/L	5.00 (4.70, 5.20)	6.00 (5.60, 6.30) ^a^	7.30 (6.80, 8.20) ^b, c^	< 0.001
HGB, mmol/L	151.00 (138.00, 161.00)	154.00 (146.00, 163.00) ^a^	156.00 (146.00, 165.00) ^b^	< 0.001
Cr, µmol/L	71.00 (58.00, 82.00)	76.00 (67.00, 85.00) ^a^	74.00 (65.00, 81.50)	< 0.001
UA, µmol/L	363.00 (298.00, 433.75)	396.00 (331.00, 457.00) ^a^	380.00 (316.25, 449.00)	< 0.001
Hypertension, n (%)	201 (12.30)	250 (29.73) ^a^	41 (37.96) ^b^	< 0.001
Dyslipidemia, n (%)	483 (29.56)	393 (46.73) ^a^	54 (50.00) ^b^	< 0.001

^a^ indicates that the Trajectory Pre-DM group is different from the Trajectory NGR group; ^b^ indicates that there are differences between Trajectory DM group and Trajectory NGR group. ^c^ indicates that the Trajectory Pre-DM group is different from the Trajectory DM group

During the follow-up, the overall atherosclerosis risk prevalence was 41.04%. In unadjusted analyses, the Trajectory Pre-DM and Trajectory DM groups showed 158% (*OR*: 2.58, 95% *CI*: 2.17–3.06) and 130% (*OR*: 2.30, 95% *CI*: 1.55–3.40) higher risks of atherosclerosis than the Trajectory NGR group. After adjusting for age, sex, BMI, SBP, DBP, heart rate, waistline, LDL-C, HGB, Cr, UA, their risks remained significantly higher (*OR*: 2.02, 95% *CI*: 1.63–2.51) for Trajectory Pre-DM, (*OR*: 1.58, 95% *CI*: 1.01–2.47) for Trajectory DM. For atherosclerosis risk incidence, the overall rate was 15.40%. The Trajectory Pre-DM and Trajectory DM groups had 130% (*OR*: 2.30, 95% *CI*: 1.71–3.09) and 184% (*OR*: 2.84, 95% *CI*: 1.43–5.67) increased risks, respectively. After full adjustment, the risk in the Trajectory Pre-DM group stayed significant (*OR*: 1.66, 95% *CI*: 1.15–2.39). The atherosclerosis risk recovery rate was 21.24%. In unadjusted models, the Trajectory Pre-DM group had a lower likelihood of atherosclerosis risk recovery (*OR*: 0.69, 95% *CI*: 0.50–0.94) than the Trajectory NGR group, and this remained significant after adjusting for metabolic factors (*OR*: 0.55, 95% *CI*: 0.39–0.79) (Table 5).

**Table 5:** Association of FBG trajectories with AIP during the three follow-up visits

Character	Unadjusted OR (95% CI)	Model1 OR (95% CI)	Model2 OR (95% CI)
Prevalence			
Trajectory NGR group	Reference	Reference	Reference
Trajectory Pre-DM group	2.58 (2.17–3.06)	2.56 (2.10–3.14)	2.02 (1.63–2.51)
Trajectory DM group	2.30 (1.55–3.40)	1.99 (1.31–3.03)	1.58 (1.01–2.47)
Incidence			
Trajectory NGR group	Reference	Reference	Reference
Trajectory Pre-DM group	2.30 (1.71–3.09)	1.94 (1.37–2.75)	1.66 (1.15–2.39)
Trajectory DM group	2.84 (1.43–5.67)	2.21 (1.06–4.61)	1.77 (0.83–3.78)
Recovery			
Trajectory NGR group	Reference	Reference	Reference
Trajectory Pre-DM group	0.69 (0.50–0.94)	0.54 (0.39–0.77)	0.55 (0.39–0.79)
Trajectory DM group	1.45 (0.83–2.55)	1.18 (0.65–2.14)	1.16 (0.63–2.15)

Model 1, logistic regression adjusted for sex and age

Model 2, logistic regression adjusted for sex and age, BMI, SBP, DBP, heart rate, waistline, LDL-C, HGB, Cr, UA

### Dose-Response relationship

**Fig. 3:**
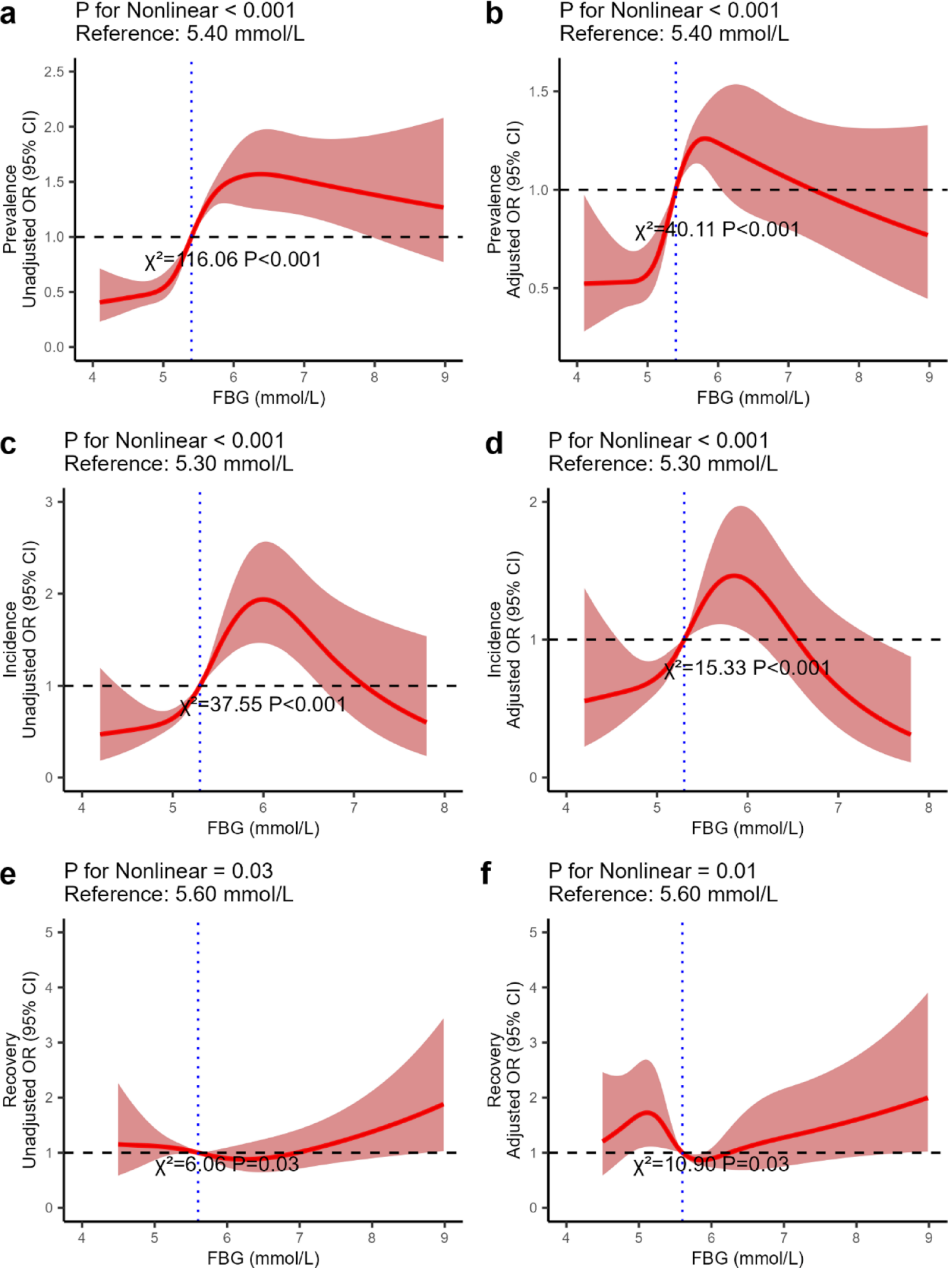
RCS Used to Visualize the Relationship Between FBG Concentration and Atherosclerosis Risk. (**a**) is the unadjusted RCS of FBG level and prevalence; (**b**) is the adjusted RCS of FBG level and prevalence; (**c**) is the unadjusted RCS of FBG level and incidence; (**d**) is the adjusted RCS of FBG level and incidence; (**e**) is the unadjusted RCS of FBG level and recovery; (**f**) is the adjusted RCS of FBG level and recovery

After defining the relationship between FBG trajectories and atherosclerosis risk prevalence, incidence, and recovery, we used RCS to model and visualize the dose-response relationship between FBG levels and atherosclerosis risk (Fig. 3). In unadjusted models, a nonlinear relationship between FBG and atherosclerosis risk prevalence was found with an inflection point, and this remained significant after covariate adjustment (*P* < 0.001). We further determined that the risk of atherosclerosis significantly increased when the FBG level was between 5.10 mmol/L and 5.90 mmol/L. Similarly, nonlinear relationships were observed between FBG and atherosclerosis risk incidence/recovery before and after covariate adjustment (*P* for nonlinearity < 0.05). When the FBG level is between 5.00 mmol/L and 5.90 mmol/L, the risk of atherosclerosis incidence increases significantly. For FBG between 5.10 mmol/L and 5.90 mmol/L, the curve *OR* for atherosclerosis recovery decreased, but when FBG exceeded 5.90 mmol/L, atherosclerosis risk recovery dropped sharply with increasing FBG.

### Subgroup analysis

**Fig. 4:**
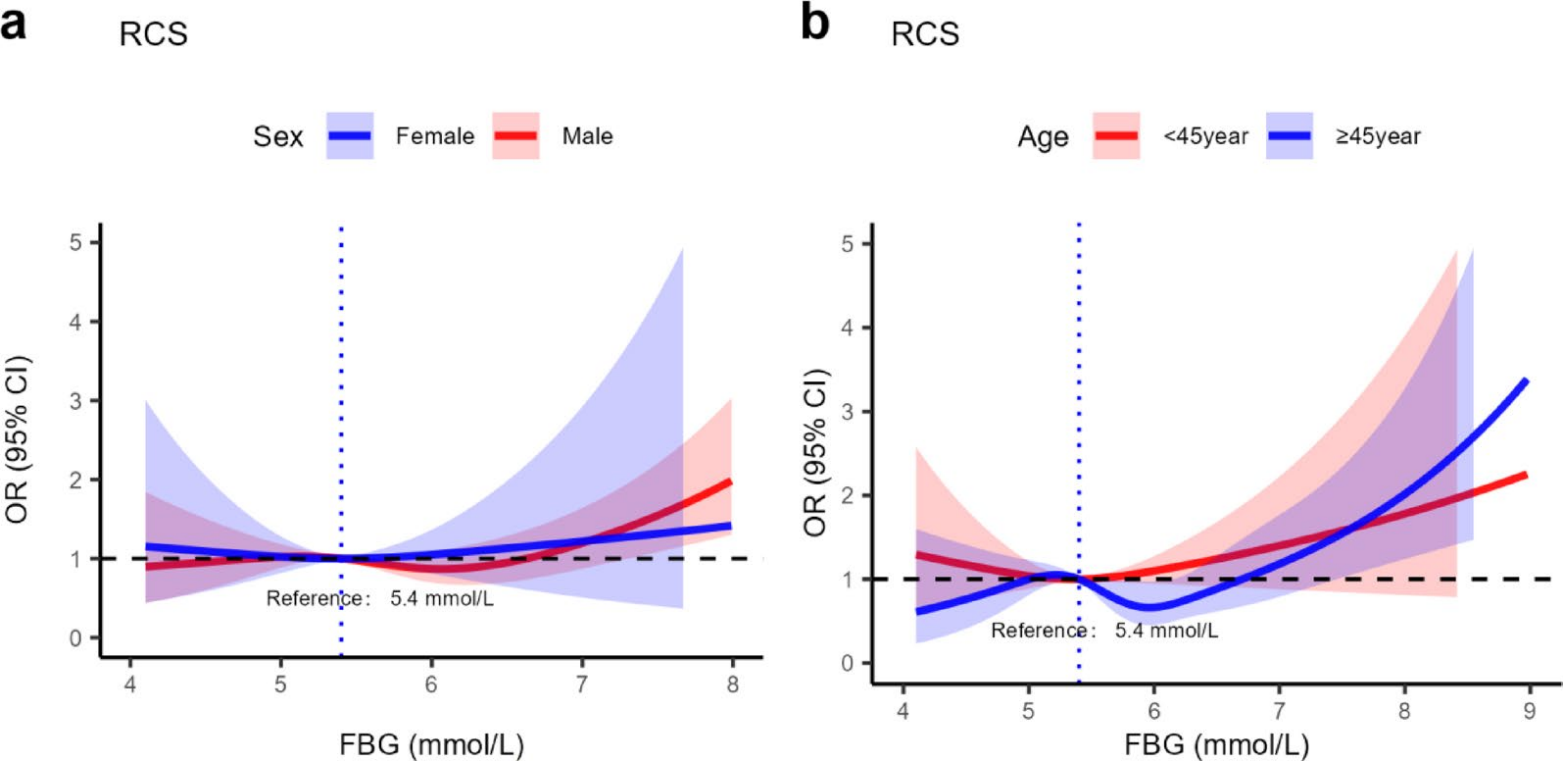
RCS analysis of the relationship between FBG trajectory and atherosclerosis risk prevalence. (**a**) shows the relationship between FBG trajectory and atherosclerosis risk prevalence by sex stratification. (**b**) shows the relationship between FBG trajectory and atherosclerosis risk prevalence by age stratification

We analyzed the association of FBG trajectories with atherosclerosis risk across different age, sex and BMI. In these subgroups, we observed interactions between sex, age and FBG trajectories in relation to atherosclerosis risk prevalence. Specifically, compared with individuals ≥ 45 years old (*OR*: 1.32, 95% *CI*: 1.07–1.62), those < 45 showed a stronger positive link (*OR*: 2.60, 95% *CI*: 1.81–3.74) between FBG trajectories and atherosclerosis risk prevalence (*P* for interaction < 0.001). Similarly, women (*OR*: 2.80, 95% *CI*: 1.89–4.13) had a stronger association than men (*OR*: 1.40, 95% *CI*: 1.16–1.67) *(P* for interaction < 0.001)(Table 6). Based on these interactions, we carried out sex and age stratification using RCS to explore the dose-response relationship between FBG trajectories and atherosclerosis risk prevalence. Figure 4a shows that in men, atherosclerosis risk rose rapidly when FBG was between 5.10 mmol/L and 5.80 mmol/L. In women, a similar significant association appeared when FBG was between 5.00 mmol/L and 6.00 mmol/L. Figure 4b reveals that in individuals aged 45 and above, atherosclerosis risk prevalence increased markedly with FBG over 5.80 mmol/L.

**Table 6:** Associations between FBG trajectories and arteriosclerosis risk by age, sex, and BMI

Character	OR (95% CI)		
	Prevalence	Incidence	Recovery
Sex			
Man (*n* = 1729)	1.40 (1.16–1.67)	1.34 (0.98–1.83)	0.99 (0.74–1.33)
Female (*n* = 854)	2.80 (1.89–4.13)	2.42 (1.32–4.41)	0.73 (0.37–1.43)
* P* for interaction	< 0.001	0.044	0.550
Age, year			
< 45 (*n* = 1186)	2.60 (1.81–3.74)	2.34 (1.25–4.38)	0.65 (0.38–1.12)
≥ 45 (*n* = 1397)	1.32 (1.07–1.62)	1.46 (1.02–2.07)	1.19 (0.84–1.68)
* P* for interaction	< 0.001	0.111	0.074
BMI, kg/m^2^			
< 24 (*n* = 1454)	1.55 (1.20–2.00)	1.88 (1.18–2.97)	1.12 (0.76–1.66)
24–27.9 (*n* = 909)	1.57 (1.24–2.00)	1.22 (0.90–1.95)	0.77 (0.51–1.18)
≥ 28 (*n* = 220)	1.44 (0.88–2.36)	1.50 (0.64–3.51)	1.25 (0.51–3.07)
* P* for interaction	0.501	0.297	0.737

## Discussion

This study provides novel insights into the dynamic relationship between FBG and atherosclerosis risk in Chongqing, China. Using longitudinal trajectory modeling, we demonstrated that elevations in FBG, even below conventional prediabetes thresholds, were independently associated with a higher likelihood of elevated atherosclerosis risk. The identification of a population-specific risk threshold, 5.10 mmol/L, lower than current international criteria, underscored the need for regionally tailored glycemic control guidelines.

The integration of cross-sectional and longitudinal analyses in our study provided robust evidence for the long-term impact of abnormalities in glucose metabolism on the risk of atherosclerosis. The cross-sectional analysis showed that both the prediabetes and diabetes groups had significantly higher atherosclerosis prevalence compared to the NGR group, indicating that elevated blood glucose at a single time point was an important risk factor for atherosclerosis [4, 42–45]. The longitudinal analysis further supported and extended on these findings. Specifically, individuals in the Trajectory Pre-DM and Trajectory DM groups exhibited a significantly higher risk of both prevalent and incident atherosclerosis. Furthermore, the likelihood of atherosclerosis risk recovery was lower in the Trajectory Pre-DM group compared to those in the Trajectory NGR group. These results highlighted the importance of identifying glucose abnormalities not only at single point but also over time, emphasizing the potential benefits of long-term glycemic control in reducing vascular risk [46, 47]. A potential pathological mechanism is that the dynamic imbalance between insulin resistance and compensatory hyperinsulinemia, which is already present in the prediabetic stage, may activate the MAPK pathway, leading to impaired endothelium-dependent vasodilation and thereby accelerating the progression of atherosclerosis risk [48, 49]. It is worth noting that although the aforementioned mechanisms may further deteriorate during the diabetic stage, the pathological processes are already initiated in the prediabetic phase [50, 51]. Moreover, even in the absence of overt insulin resistance, fluctuations in blood glucose levels can activate the neutrophil–S100A8/A9–RAGE axis, which promotes myelopoiesis in the bone marrow, increases circulating inflammatory monocytes, and enhances infiltration into the vascular wall, thereby contributing to an increased atherosclerosis risk [52]. In addition, oxidative stress induced by hyperglycemia, along with the excessive generation of reactive oxygen species, has been shown to further compromise the capacity for vascular repair [53–57]. Beyond biological mechanisms, social cognitive biases may further aggravate vascular risk in individuals with prediabetes, since it is often misconstrued by both the public and healthcare professionals as a mild or transitional state below the threshold of overt disease [49]. This misperception often results in an underestimation of its vascular implications, leading to insufficient implementation of preventive strategies such as dietary control and physical activity [58]. As a result, early glycemic fluctuations may be overlooked, allowing metabolic disturbances that could have been reversed through timely behavioral modification to progress unchecked [59–62], ultimately creating a synergistic effect with insulin resistance, MAPK pathway activation, and oxidative stress, to reduce the likelihood of atherosclerotic risk recovery [48, 49, 52–57]. Therefore, by enhancing risk awareness, promoting early intervention, and ensuring dynamic monitoring, targeted management strategies for prediabetes can be developed to effectively disrupt the vicious cycle by which metabolic abnormalities advance to endothelial dysfunction and vascular injury [50].

Our study also examined the dose-response relationship between FBG levels and both the prevalence and incidence of atherosclerosis risk in the Chongqing population. The thresholds for atherosclerosis risk prevalence and incidence in the Chongqing population were 5.10 mmol/L and 5.00 mmol/L, respectively, which are significantly lower than the ADA’s prediabetes standard of 5.60 mmol/L [33]. This finding suggested that reliance on the ADA diagnostic criteria may lead to underestimation of early vascular injury in the prediabetic population in Chongqing, China. Notably, even within the conventionally defined normal range, from 5.00 to 5.60 mmol/L, individuals already exhibited a significantly elevated risk of atherosclerosis. The observed lower threshold in our cohort may be attributable to a combination of regional genetic background and dietary habits of the Chongqing population. Cui et al. suggested that genetic polymorphisms related to glucose metabolism may exist in the Chinese population, resulting in increased sensitivity to elevated blood glucose levels [63].A prospective cohort study by Miller et al., which included 127,594 participants from 20 countries, found that the glycemic index of the Chinese diet was the highest, at 88.9 [64]. Compared with diets with a lower glycemic index, high–glycemic index diets were associated with a 15% increased risk of developing diabetes [64]. Zhang’s research indicates that under the influence of regional climate, the southwestern region of China has long maintained a high-GI diet characterized by high oil and high salt intake [65, 66]. Wu et al. conducted abdominal CT scans in 485 participants from Xiamen, China, to measure visceral fat area and validated their findings in an independent cohort of 6,495 participants from Shanghai, China [67]. They found that the China Visceral Adiposity Index they developed was strongly associated with both visceral obesity and insulin resistance, suggesting that individuals in the Chinese population are more prone to visceral fat accumulation, which may lead to metabolic disturbances even at relatively low FBG levels [67].These findings implied that the ADA diagnostic criteria might have limitations in identifying atherosclerosis risk in the Chongqing population. Thus, atherosclerosis risk assessment strategies in this region should not simply apply the ADA diagnostic thresholds but rather consider local population characteristics to develop more precise screening criteria and intervention strategies for earlier detection and management of atherosclerosis risk.

Our subgroup analyses further revealed significant sex- and age-related differences in the association between FBG levels and the risk of atherosclerosis in the Chongqing population The atherosclerosis risk threshold was identified as 5.00 mmol/L for females and 5.10 mmol/L for males. Peters et al. demonstrated that female patients with diabetes have a significantly higher risk of stroke compared to male patients [68], and Wang et al. research further showed that, relative to men, women with diabetes have a 58% higher risk of coronary heart disease and a 13% higher risk of all-cause mortality [69], suggesting that women may exhibit greater pathophysiological sensitivity to elevated blood glucose levels. Furthermore, this study was the first to demonstrate that the atherosclerosis risk threshold was significantly lower in individuals under 45 years of age (5.00 mmol/L) compared to those aged 45 years and older (5.80 mmol/L). This age-specific discrepancy in risk thresholds suggests that younger individuals may be more susceptible to atherosclerosis development at comparatively lower FBG levels. This finding is consistent with trends reported by the Global Burden of Disease study, which has shown a shift toward younger age at onset of cardiovascular diseases in recent years, particularly among individuals with overweight or prediabetes [70, 71]. This phenomenon is particularly pronounced in China, where the younger generation has grown up during a period of rapid socioeconomic development and has been chronically exposed to high-calorie diets, sedentary lifestyles, and sustained metabolic stress, which may contribute to reduced insulin sensitivity, thereby accelerating the onset of metabolic disorders and increasing atherosclerosis risk at an earlier age [72, 73]. In contrast, older adults in China were born before The 1980 s, a period characterized by higher levels of physical activity and simple, unprocessed diets [74, 75]. Moreover, many older individuals continue to follow traditional health practices, such as regular physical activity and dietary restraint, which may further support vascular integrity even in the presence of higher FBG levels [76, 77]. This phenomenon may be partly explained by the concept of metabolic memory, which suggests that early exposure to hyperglycemia can induce persistent epigenetic modifications and inflammatory responses at the cellular level, thereby sustaining an elevated cardiovascular risk even after subsequent improvements in glycemic control [78, 79]. These findings underscore the population-specific impact of glucose metabolic dysregulation on the vascular system and further support the need for individualized intervention strategies tailored to different age and sex groups. In particular, early identification and management of mild hyperglycemia may be especially important in women and younger individuals to prevent the development of cardiovascular events.

Based on these findings, we propose an atherosclerosis risk prevention and control strategy centered on FBG management. First, clinical practice should shift from relying solely on single-point blood glucose evaluations to emphasizing long-term FBG trajectories for early warning and atherosclerosis risk prevention. Second, we recommend setting the primary prevention threshold for atherosclerosis risk at 5.00 mmol/L in the Chongqing population, with dynamic monitoring protocols for individuals whose FBG levels fall within the 5.00–5.60 mmol/L range. Third, sex- and age-specific intervention strategies should be developed, incorporating multidimensional approaches for individuals with prediabetes, including lifestyle modification, pharmacological treatment, and continuous metabolic surveillance. Clinical practice should move beyond conventional diagnostic thresholds and adopt a region- and population-specific FBG management framework to more effectively mitigate early vascular damage and slow the progression of atherosclerosis risk.

### Strength and limitation

This study has several strengths, including its use of longitudinal data, which allowed for the observation of the dynamic relationship between FBG changes and atherosclerosis risk. We also performed dose-response and subgroup analyses to identify specific thresholds for atherosclerosis risk and to explore the effects of demographic factors on atherosclerosis outcomes. However, there are several limitations. First, a three-year follow-up period may not be sufficient to fully capture the metabolic memory effect of long-term fluctuations in FBG on the progression of atherosclerosis risk. Longer-term cohort studies are needed to confirm our findings. Second, the data were derived from a single center in Chongqing, which may limit the generalizability of the results to other populations, particularly those with vastly different genetic backgrounds, diets, and lifestyles. The FBG thresholds identified here should therefore be considered primarily relevant to the Chongqing population and similar regional cohorts unless validated externally. Third, as an observational study, causal inference cannot be established, and residual confounding or reverse associations between FBG and AIP may still exist despite longitudinal analyses and multivariable adjustments. Fourth, it is important to note that AIP was selected as a cost-effective and feasible surrogate marker for atherosclerosis risk in this large-scale health check-up cohort, rather than a direct measure of atherosclerotic plaque burden [24, 35]. While this approach enhances practicality and reproducibility, it does not replace the diagnostic value of established imaging-based markers such as carotid intima-media thickness or coronary artery calcium score. Future studies that integrate these direct imaging markers alongside AIP would provide a more comprehensive assessment of vascular health. Fifth, the GBTM used in this study assumes that individuals within each trajectory group follow exactly the same variation pattern, without considering the variations among individuals within the group. Although this method is helpful for identifying representative macroscopic trajectories, it may simplify the true heterogeneity of blood glucose changes.

## Conclusion

In summary, this study demonstrates that individuals in the Chongqing region exhibit elevated atherosclerosis risk at relatively low FBG levels, emphasizing the importance of early intervention during the prediabetes stage. Only through early and precise identification coupled with multidimensional interventions can the onset and progression of atherosclerosis risk be effectively prevented and delayed, thereby providing a more scientific and personalized approach to managing glucose metabolism abnormalities. Future research should further explore the underlying mechanisms linking glucose metabolism abnormalities and atherosclerosis risk and evaluate the efficacy of personalized intervention strategies across different subgroups to reduce the public health burden of atherosclerosis.

## Supplementary Information

Supplementary Material 1

## Data Availability

The data of the current study are available from the corresponding author on reasonable request.

## Abbreviations

ADA: American Diabetes Association
AIC: Akaike Information Criterion
AIP: Atherogenic index of plasma
APPA: Average posterior probability of assignment
BIC: Bayesian Information Criterion
BMI: Body mass index
DM: Diabetes mellitus
FBG: Fasting blood glucose
GBTM: Group - based trajectory model
HDL-C: High-density lipoprotein cholesterol
HGB: Hemoglobin
IQR: Interquartile ranges
LDL-C: Low-density lipoprotein cholesterol
NGR: Normal glucose regulation
Pre-DM: Prediabetes mellitus
RCS: Restricted cubic splines
Cr: Serum creatinine
TC: Total cholesterol
TG: Triglycerides
UA: Uric acid
WHO: World Health Organization
