# Supplementary material for: Fasting blood glucose trajectories and atherosclerosis risk: a longitudinal cohort study with threshold analysis in Chongqing, China
Source: J Health Popul Nutr. 2026 Mar 5;45:103. doi: 10.1186/s41043-025-01193-7 (PMC13020111; doi:10.1186/s41043-025-01193-7)
Supplement: Supplementary file 1 — Supplementary Material 1 [file 41043_2025_1193_MOESM1_ESM.docx]

**SUPPLEMENTARY MATERIAL**

**Title: Fasting Blood Glucose Trajectories and Atherosclerosis Risk: A Longitudinal Cohort Study with Threshold Analysis in Chongqing, China**

**Contents**

**Fig. S1** Power analysis using GPower.

**Table S1** Model Performance of Trajectory Models


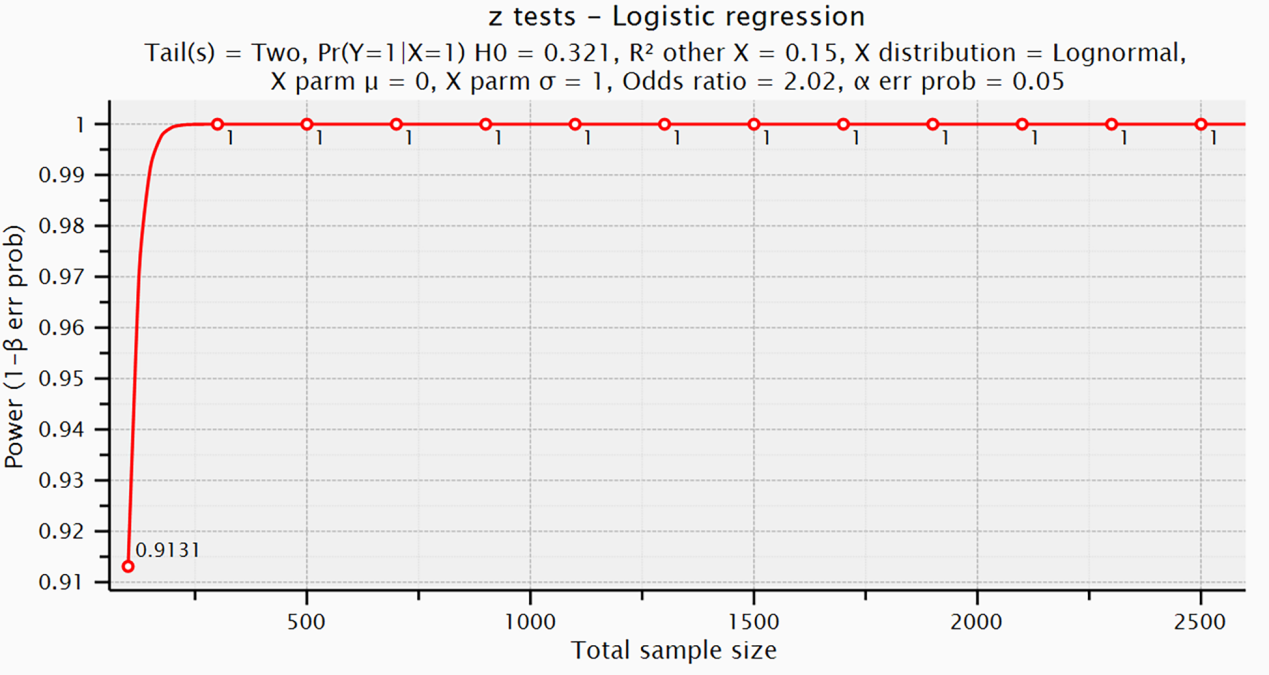


Figure S1. Post-hoc power analysis for logistic regression. The curve displays the relationship between statistical power (y-axis) and total sample size (x-axis).

**Table S1** Model Performance of Trajectory Models

| Group | Percent (%) | BIC^#2^ | △BIC^#2^ | AIC | △AIC | Avepp (%) | Ek |
| --- | --- | --- | --- | --- | --- | --- | --- |
| 1Group (1) | 100.00 | -10147.94 |  | -10137.46 |  | 100.00 | 0.000 |
| 2Group (1 1) | 86.11: 13.89 | -8961.27 | 1186.68 | -8940.29 | 1197.16 | 97.72: 92.16 | 0.891 |
| 3Group (1 1 1) | 63.11: 32.56: 4.33 | -8445.00 | 516.27 | -8413.54 | 526.76 | 95.80: 88.55: 94.29 | 0.852 |
| 4Group (1 1 1 1) | 19.53: 44.25: 31.73: 4.49 | -8458.49 | -13.49 | -8416.54 | -3.00 | : : : | 0.599 |
| 5Group (1 1 1 1 1) | 17.81: 24.82: 21.15: 31.73: 4.49 | -8471.97 | -13.49 | -8419.54 | -3.00 | : : : : | 0.467 |
| 1Group (2) | 100.00 | -10152.44 | . | -10138.46 | . | 100.00 | 0.000 |
| 2Group (2 2) | 86.11: 13.89 | -8970.06 | 1182.38 | -8942.10 | 1196.36 | 97.73: 92.14 | 0.891 |
| 3Group (2 2 2) | 63.22: 32.49: 4.29 | -8457.65 | 512.41 | -8415.70 | 526.40 | 95.74: 88.64: 94.56 | 0.853 |
| 4Group (2 2 2 2) | 16.69: 47.11: 31.72: 4.48 | -8475.63 | -17.98 | -8419.70 | -4.00 | . : . : . : . | 0.619 |
| 5Group (2 2 2 2 2) | 18.62: 23.92: 21.27: 31.72: 4.48 | -8493.62 | -17.98 | -8423.70 | -4.00 | . : . : . : . : . | 0.466 |
| 1Group (3) | 100.00 | -10156.89 | . | -10139.41 | . | 100.00 | 0.000 |
| 2Group (3 3) | 86.11: 13.89 | -8978.33 | 1178.56 | -8943.37 | 1196.04 | 97.75: 92.11 | 0.891 |
| 3Group (3 3 3) | 63.22: 32.49: 4.29 | -8470.97 | 507.36 | -8418.53 | 524.84 | 95.75: 88.61: 94.55 | 0.853 |
| 4Group (3 3 3 3) | 29.25: 34.57: 31.71: 4.48 | -8493.45 | -22.48 | -8423.53 | -5.00 | . : . : . : . | 0.566 |
| 5Group (3 3 3 3 3) | 26.16: 24.00: 13.66: 31.71: 4.48 | -8515.93 | -22.48 | -8428.53 | -5.00 | 188.39:: . : . : . | 0.478 |
| Note: AIC (Akaike information criterion), BIC#2(Bayesian Information Criterion), Ek(Relative entropy), Avepp (Average posterior probability) | | | | | | | |
